# Wounds and Bruises

**Published:** 1873-02

**Authors:** 


### WOUNDS AND BRUISES

May be often cured in a very. short time by the use of arnica or veratrum viside, which can be had at any good drug store in the form of a tincture. All that is necessary to be done is to apply it lightly with a mop or the finger, or by dipping a cloth or some lint into it and placing it on the injured part; it causes inflammation with some stinging and burning. Twenty grains mixed with an ounce of lard makes a very convenient ointment to be kept on hand in every household; the more familiar name is the poke berry root. Take eight ounces of the dried root and let it soak for two weeks in double the amount of alcohol—that is, sixteen ounces or one pint. This forms the tincture. The ointment is made by mixing forty grains of the powder with an ounce of lard. The preparations of arnica are better, but the poke root can be so generally had in many parts of the country that it is mentioned first. But as aconite is one of the most valuable of all the drugs, it should be used in preference when it can be had; the common name is monks-hood. The fresh leaves and flowers should be gathered when the blossom is about one-third expanded, the root should be gathered in the winter or early spring; eight ounces of the root in a pint of alcohol makes the tincture.

But persons may have wounds and bruises many miles away from these useful articles: a good substitute is to cut up some leather in small pieces and lay it on burning coals, holding the injured part in such a way that the smoke may reach it; hold it in the smoke a few minutes and repeat it two or three times a day: this has cured old sores of very long standing in a short time when the bowels have been regularly active once a day, and the person lives on fruit, berries and coarse bread.

